# 

**Published:** 1894-11-10

**Authors:** 


PRICE TWOPENCE.

No. 424, Vol. XVII.—Nov. 10th, 1894.

[Registered at the General Post Office as a Newspaper for transmission in the United Kingdom and Abroad.]

WITH EXTRA NURSING SUPPLEMENT.

Per Annum, 10s. 6d., post free.

Monthly Parts, 6d.; post free, 8d.

Ditto per Annum, 8s. 6d. post free.

### THE HOSPITAL

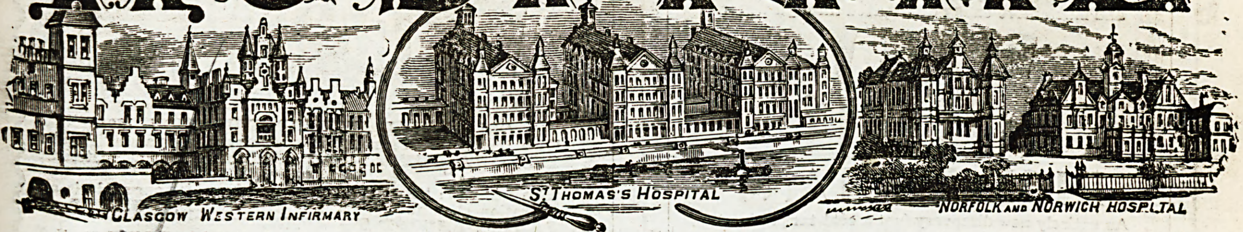

No. 424. Vol. XVII. WITH EXTRA NURSING SUPPLEMENT. SATURDAY, NOV. 10TH, 1894.

